# Clusters of oculocutaneous albinism in isolated populations in Brazil: A community genetics challenge

**DOI:** 10.1590/1678-4685-GMB-2023-0164

**Published:** 2023-12-18

**Authors:** Paulyana Moura, Augusto César Cardoso-dos-Santos, Lavinia Schuler-Faccini

**Affiliations:** 1 Universidade Federal do Rio Grande do Sul, Instituto de Biociências, Programa de Pós-graduação de Genética e Biologia Molecular, Porto Alegre, RS, Brazil. Universidade Federal do Rio Grande do Sul Instituto de Biociências Programa de Pós-graduação de Genética e Biologia Molecular Porto Alegre RS Brazil; 2 Ministério da Saúde, Governo Federal, Brasília, DF, Brazil. Ministério da Saúde Governo Federal Brasília DF Brazil; 3 Instituto Nacional de Ciência e Tecnologia de Genética Médica Populacional (INaGeMP), Porto Alegre, RS, Brazil. Instituto Nacional de Ciência e Tecnologia de Genética Médica Populacional Porto Alegre RS Brazil; 4 Hospital de Clínicas Porto Alegre HCPA, Porto Alegre, RS, Brazil. Hospital de Clínicas Porto Alegre HCPA Porto Alegre RS Brazil

**Keywords:** Albinism, isolated populations, founder effect, inbreeding, narrative review

## Abstract

Oculocutaneous albinism (OCA) is a heterogeneous group of genetic disorders involving deficiencies in melanin biosynthesis, with consequent skin, hair, and eye hypopigmentation. The world prevalence is estimated at 1/17,000, but there is high variability among populations. The affected individuals, besides clinical complications, can suffer from discrimination. The Brazilian population is highly admixed, with isolated and inbred communities. Previous reports indicated the presence of diverse isolated communities with a high prevalence of OCA in Brazil. The present work sought to review and characterize clusters of albinism in this country based on scientific literature search, newspapers, and websites. We identified and characterized 18 clusters, 13 confirmed by scientific studies. Seven clusters are in the Northeast region, with predominant African ancestry, and seven others in indigenous communities, particularly among the Kaingaing in South Brazil. Isolation and inbreeding associated with founder effects seem to be the most plausible explanation. Molecular studies and clinical classification are still limited. Their localization in deprived regions with poor infrastructure makes them particularly vulnerable to the social and clinical consequences of lacking melanin. We reinforce the need for a tailored approach to these communities, including appropriate medical care, social support, and genetic counselling.

## Introduction

### Defining albinism: clinical types and genes

Albinism is a term that refers to a heterogeneous group of congenital genetic disorders involving deficiencies in the metabolic route of melanin biosynthesis. As a result, the individuals present various degrees of hypopigmentation of the skin, hair, and eyes (Liu et al., 2021). Besides the iris and retinal hypopigmentation, ocular manifestations include congenital nystagmus, reduced visual acuity, refractive errors, photophobia, abnormalities in the optic nerves and strabismus. There is also an increased risk of skin cancer (Grønskov and Brondum-Nielsen, 2007).

Different forms of albinism were firstly classified according to the clinical characteristics and mode of inheritance. The most frequent phenotypes are related to non-syndromic, autosomal recessive oculocutaneous albinism (OCA). In its most severe form, OCA1, melanin production can be completely absent. OCA1 is caused by mutations in the Tyrosinase gene (*TYR*). At least seven other types of OCA (OCA2 to OCA8) were also described, genetically related to different genes associated with melanocyte differentiation, melanosomal proteins and melanin synthesis (Fernández et al., 2021).

Less frequent are the ocular albinism types (OA), with manifestations restricted to the eyes and X-linked inheritance and the syndromic forms of albinism, associated with other systemic manifestations, such as immunological deficiencies, pulmonary fibrosis and hematological conditions. Hermansky-Pudlack and Chediak-Higashi syndromes are the best-known examples of syndromic albinism (Scheinfeld, 2003).

### Historical notes

Ancient reports of albinism are traced back to the Bible, where the description of Noah suggests that he could be a person with this condition. The term refers to the word “white”, which comes from the Latin (*albus*). However, more consistent descriptions of people with phenotypes resembling OCA were reported during the period of the great navigations. The term “albino” was apparently first used by Balthazar Tellez, a Jesuit priest, in his chronicles of the Jesuit mission to Ethiopia during the 1600s, later translated to English and printed in London (Teles, 1710). In the early 20^th^ century, Sir Archibald Garrod identified albinism as an inborn error of metabolism secondary to an enzymatic defect in melanin synthesis (Teles, 1710; King,1987). 

### Prevalence around the world

The worldwide prevalence of all forms of OCA was estimated at 1:17,000 by Witkop et al. (1989). Although this is an old publication, it is still generally referred to by most authors (Ramos et al., 2021). Studies at the country level show that OCA1 is more prevalent in white populations, while OCA2 is predominant in African countries. In Europe, estimates range from 1:10,000 in Ireland (Froggatt, 1960) to 1:15 000 in the Netherlands (Van Dorp, 1987). In the USA, the prevalence for the black population was 1:10,000, whilst that for the white population was 1:19,000 (Witkop *et al.*, 1989). In South Africa, albinism affects about 1:4000 people, and in Nigeria, 1:5,000 (Hilton, 2021; Kromberg and Kerr, 2022). High prevalences are also reported in groups with high consanguinity, such as the Bhatti Tribe in Pakistan (5 in 100 people), and among indigenous communities, for example, in Kuna in Panama, where the ratio is 1:200, similar to the rate of Hopi people in North America, with a prevalence of 1:227 (Woolf and Grant, 1962; Keeler, 1964). In Brazil, although the nationwide prevalence is not available, reports mention communities with a higher prevalence of albinism both in indigenous (Salzano, 1961) and in admixed populations (Freire-Maia et al., 1978). Around 1,000 Brazilian individuals are estimated to have albinism (Marçon et al., 2020).

### Myths, discrimination, ethics, public policies

People with albinism constantly suffer from stigma and prejudice about their condition, which represent barriers to the right to access health, education, and citizenship and prevent the full inclusion of people with albinism in society. In areas with a high incidence of albinism, such as sub-Saharan Africa, there are many myths and beliefs related to protection or luck linked to the presence of people with albinism, which leads to their persecution, attacks, maiming and murder (Brocco, 2016). 

In the Americas, cultural selection for people with albinism was also described. In the Kuna original people in Panamá, marriage discrimination against albino males and infanticide was described (Woolf, 2005). On the other side, in the Hopi native people in Arizona, people with albinism were well integrated and had religious or social privileges, which was suggested as a mechanism of positive sexual selection (Woolf and Grant, 1962)

In Brazil, the social and psychological vulnerabilities faced by individuals with albinism are not yet fully known. Still, studies have pointed to a strong burden of stigma and prejudice, which adds to the uneven distribution of other social and health vulnerabilities in its territory (Maia et al., 2015; Marçon et al., 2020; Brasil, 2021). This situation tends to be even more sensitive in geographically isolated areas with a high frequency of albinism, considering that clusters of genetic conditions (especially conditions with a visible, disfiguring phenotype) are places constantly surrounded by myths and beliefs and where there is not always all the necessary health services for comprehensive, timely health care (Pereira, 2005; Arruda et al., 2020; Cardoso-dos-Santos et al., 2020). 

Such particularities highlight albinism as a public health issue. In Tanzania, for example, Regional Dermatological Training Center runs a mobile skin care clinic where a doctor and a nurse regularly visit villages to check the skin of people with albinism and provide education on protection from UV exposure (Brocco, 2016). 

Countries have been urged to combat attacks and discrimination against people with albinism. In 2013, the Human Rights Council of the United Nations General Assembly established that countries should “take all necessary measures to ensure the effective protection of persons with albinism and their family members”. In addition, the Resolution encourages countries to share best practices in protecting and promoting the rights of persons with albinism (United Nations, 2013). 

In Brazil, some initiatives at the federal level aim to promote health care for people with albinism, including funding for equity actions in Primary Health Care, considering people with albinism (Brasil, 2020). However, many individuals arrive at the health system only when there are severe complications, such as neoplasia or visual deficiency (Brasil, 2021). 

## Clinical and molecular characterization of albinism in Brazil

Few published studies describe the molecular profile of Brazilian cases of albinism. Ribeiro (2019) compared clinical and ophthalmological characteristics with the molecular results obtained from sequencing the *TYR* and *OCA2* genes in 21 patients from São Paulo. Three patients were identified with a pathogenic variant in OCA1 and 18 in OCA2 (2019). Through the dermatological evaluation only, the author classified six patients as having OCA1 and 15 as having OCA2, reinforcing the importance of genetic tests for the correct diagnosis. Schidlowski et al. (2020) performed whole exome sequencing on eight children with a clinical diagnosis of OCA in Paraná state. In five, they identified pathogenic variants in the gene *TYR* (OCA1), three compound heterozygous with one novel variant. One individual with a variant in the gene *SLC45A2* (OCA4) is also a compound heterozygous. Although limited in sample size, these two studies suggest significant variability in the genetic architecture of albinism in Brazil.

Moreira et al. (2021) studied families in the State of Bahia based on records from the Federal University of Bahia and the Association of People with Albinism of Bahia (APALBA). Among the 457 people, 265 (58%) had a familiar recurrence. Couples composed of both albino parents produced offspring with the same condition, suggesting that the pathogenic variants were present in the same gene responsible for albinism, but molecular analyses were unavailable. 

### Albinism and the Graduate Program of Genetics and Molecular Biology (PPGBM) - UFRGS

One of the founders of studies in the genetic structure of Brazilian populations was Francisco Mauro Salzano, also a founder of PPGBM-UFRGS. One of his first internationally published papers was entitled “Rare conditions among Caingang Indians” (Salzano, 1961), where he described cases of albinism in these communities. Almost five decades later, in 2008, the National Institute of Science and Technology for Population Genetics (Instituto Nacional de Genética Médica Populacional or INAGEMP) was created. 

PPGBM-UFRGS is part of the INAGEMP, where a register of isolated populations was implemented in 2009 under the name CENISO (Portuguese acronym for National Census of Isolates; “Censo Nacional de Isolados”). CENISO is a surveillance system of sub-populations with higher-than-expected genetic or congenital conditions (geographic clusters) based on the systematic collection, recording and validation of reports - scientific or lay - of these sub-populations (Castilla and Schuler-Faccini, 2014). In an initial evaluation from CENISO, published in 2019, 12 independent reports of confirmed or probable albinism clusters were registered among 279, or 4,3% (Cardoso *et al.*, 2019).

### Motivation and objectives

The Brazilian population is highly admixed and with significant genetic African influence (Pena et al., 2011) and with the existence of isolated and consanguineous communities, especially “quilombolas” and indigenous (Cardoso-dos-Santos et al., 2020). Moreover, the CENISO database suggested that clusters of albinism in Brazil was not an uncommon occurrence. Therefore, albinism may represent an important public health topic in Brazil, both in prevalence and issues related to access. The present work sought to review and characterize clusters of albinism in this country.

## Methodology

This narrative review is based on a scientific literature search and on newspapers and websites. Scientific literature was searched in PubMed, Google Scholar and Scielo, both in English and Portuguese. The search entries were: *albinism*, Brazil, *rumor*, *founder effect*, *cluster*, *inbreeding* and *consanguineous marriages*, and its counterparts in Portuguese. 

The search through websites and news was performed on Google with the following keywords in Portuguese: *albinismo* OR *albin*o AND *Brasil*. We also made a Google search using the names of towns or communities already registered on CENISO. Another search included the keywords *albinismo* OR *albin*o AND *indigena* OR *quilombo* OR *quilombola*. The filtering was manual and considered relevance as location, number of inhabitants, number of occurrences of albinism, and type of community in addition to the search for scientific references. Reports without mention of inhabitants with albinism, or who had the first name “Albino” detected, or duplicates, were excluded.

## Results

**Figure 1 -:**
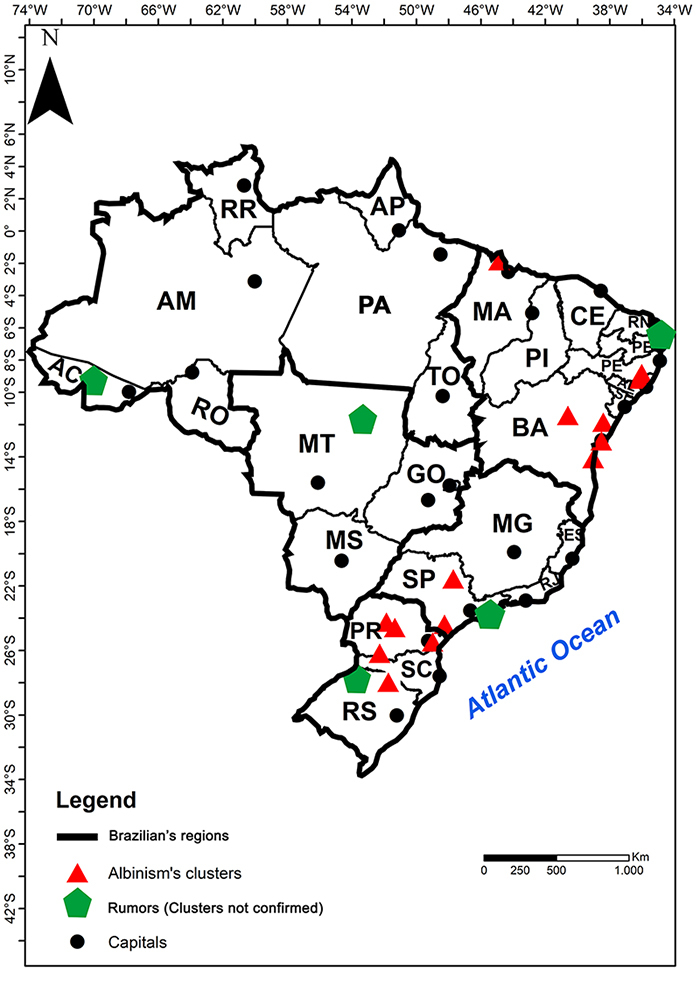
Map of Brazil with the Clusters of Albinism (AC - Acre, AL - Alagoas, AM - Amazonas, AP - Amapá, BA - Bahia, CE - Ceará, DF - Distrito Federal, ES - Espírito Santo, GO - Goias, MA - Maranhão, MG - Minas Gerais, MS - Mato Grosso do Sul, MT - Mato Grosso, PA - Pará, PB - Paraíba, PE - Pernambuco, PI - Piauí, PR - Paraná, RJ - Rio de Janeiro, RN - Rio Grande do Norte, RO - Rodônia, RR - Roraima, RS - Rio Grande do Sul, SC - Santa Catarina, SE - Sergipe, SP - São Paulo, TO - Tocantins).

The preliminary survey in the CENISO database identified 12 entries of geographic clusters of albinism in Brazil. Two registers in the CENISO were excluded since it was duplicated data (1) or unspecified geographic locations (1), and the remaining were confirmed in the scientific literature through published papers, master dissertations, monographs, and one congress abstract (Table 1). In our present review, we confirmed the nine clusters in CENISO and added four new ones, also confirmed by scientific research (in Paraná, South Brazil). Other additional four *rumors* (clusters not confirmed yet) were also detected in the grey literature: three were only from newspapers (Acre, São Paulo, Rio Grande do Sul), and one additional case report in Parque do Xingu (Freitas *et al.*, 2005) (Figure 1). Notably, clusters or rumors were predominantly reported in Indigenous areas or in communities of known African ancestry. We will present it in separate sections.

### The Indigenous communities in South Brazil

Of the 18 clusters we could locate, half were indigenous communities, and five were in the Kaingang lands (Table 1). Back in 1961, Salzano reported seven individuals with albinism in a population of about 193 individuals in two communities of the Kaingang indigenous communities in the north part of Rio Grande do Sul state (South Brazil), therefore with a prevalence of 1/28. Reports of other individuals with albinism in Kaingang communities were later reported, particularly in Paraná state. The Kaingang live on more than 30 Indigenous lands in four states in southern Brazil (São Paulo, Paraná, Santa Catarina, and Rio Grande do Sul), with around 35,000 people living in these communities (Siasi and Sesai, 2014). We also found one report of albinism in a Guarani community in Paraná. Three additional reports on indigenous communities were only reported in newspapers and limited to one case report in the Kiukuro community (Parque Xingu, Mato Grosso), one in the North (Kaxinawa, Acre) in one in the Guarani community of Ribeirao Silveira (São Sebastião, São Paulo). 

**Table 1 -:** Clusters and Rumors identified in Brazil.

Cluster / Municipality (State)	Reported cases	Population	Estimated Prevalence	Geography/ Ancestry	References
Ilha da Maré, Salvador (BA)	10	6,434	1:643	Island/ Quilombo^*^	Moreira et al. (2016)
Ilha dos Sapinhos, Maraú (BA)	5	100	1:20	Island / Diverse	Moreira et al. (2019)
Miguel Calmon (BA)	13	25,771	1:1,982	Diverse	Moreira et al. (2019)
Aldeia Lagoa Branca / Inhambupe (BA)	22	36,290	1:1,649	African descendants	Moreira et al. (2019)
Comunidade Filús, Santana do Mundaú (AL)	10	170	1:17	Quilombo^*^	Cardoso-dos-Santos et al. 2020
Ilha dos Lençóis, Cururupu (MA)	11	500	1:45	Island / Diverse	Freire-Maia et al. (1978)
Quipapá (PE)	13	26,309	1:2,023	Quilombo^*^	Cardoso-dos-Santos et al. 2020
Baía Formosa (RN)	-	9,373	-	Diverse	CENISO
Vale do Ribeira / Eldorado (SP)	5	15,000	1:3,000	Quilombo^*^	Kimura and Mingroni-Netto (2021)
Cacique Doble / Cacique Doble (RS)	8	193	1:24	Kaingang	Salzano (1961)
TI Faxinal / Candido Abreu (PR)	4	500	1:125	Kaingang	Buratto (2010)
TI Ivaí / Manoel Ribas (PR)	5	1,687	1:337	Kaingang	Buratto (2010)
TI Palmas / Palmas (PR)	3	781	1:260	Kaingang	Rodrigues (2021)
TI Caruguá, Piraquara (PR)	4	54	1:13	Mbya-Guarani	Alves (2006)
TI Alto Rio Purus / Sta Rosa do Purus (AC)	3	1,871	1:623	Kaxinawa	Newspaper
TI Ribeirão Silveira / São Sebastião (SP)	1	474	1:474	Guarani	Newspaper
Parque Indigena Xingu (MT)	1	6,090	1:6,090	Kuikuro	Freitas *et al.*, 2005 (case report)
TI Guarita / Tenente Portela (RS)	3	5,996	1:1,998	Kaingang	Newspaper

^*^Quilombos are small communities, most related to the African enslaved and their descendants.

### The “Enchanted Island”: Ilha dos Lençóis

Freire-Maia and colleagues studied an isolated community of 304 individuals on an island in the state of Maranhão and identified 18 individuals born with albinism (Freire-Maia *et al.*, 1978). White sand dunes and pristine lagoons mark the Lençóis region. Albino people in this region became known in the media as “Filhos do Rei Sebastião” or “Filhos da Lua”. The Ilha dos Lençóis is considered an “enchanted island” in the local mythology. According to a legend, Dom Sebastião, a young king of Portugal, was not killed in the famous battle of Alcacer-Quibir (Morocco) in 1578, but transported with all his court, through a sortilege of the Moorish to an island with many sand dunes, like those in Morocco, for the eternity. The appellation “Sons of the moon” is also related to the white of the sand and the aversion to the sun. Although it may sound harmless, these people report that these terms were imposed on them and increasingly reinforced the stigma associated with their physical appearance (Pereira, 2005).

### The African ancestry and the *“quilombola”* communities

One paper published in 2019 reported 34 municipalities in the state with an estimated prevalence of over 1/10,000 (ranging from 1.04 to 6.7/10,000). This is a review of records from the Association of Albinism in Bahia (Associacao de Albinos da Bahia; APALBA) (Moreira et al., 2019). Two of these municipalities were reported independently in the CENISO: Maraú (Ilha de Sapinhos) and Miguel Calmon. In the CENISO, we also found “Ilha da Maré”, an island part of Salvador, the capital of Bahia state (Moreira *et al.*, 2016; Cardoso-dos-Santos et al., 2020). We also kept another cluster reported by Moreira *et al.* (2019), Aldeia Lagoa Branca (Inhambupe, Bahia). 

Four clusters occurred in quilombo communities, that is, communities formed from the 16th century onwards due to the escape and isolation of enslaved people, mainly of African origin, and their descendants, many of whom remain in relative isolation until today (Cardoso-dos-Santos et al., 2020; Kimura, 2021). Lay and scientific literature have reported albinism among families from quilombos in Santana do Mundaú (Alagoas) (Rodrigues, 2011; Maia et al., 2015; Levy, 2019), around 70 km away from Quipapa (Pernambuco), where a rumor of albinism was registered in CENISO (Cardoso *et al.*, 2019). 

“Ilha da Maré” is considered the location with the highest percentage of black ethnicity (up to 93%) and the highest prevalence of albinism (1:1,000 inhabitants) in Salvador. Albinism in “Ilha da Maré” is reportedly concentrated in a small *quilombola* community with 400 to 500 inhabitants, with a tradition of consanguinous marriages (Moreira et al., 2016). The community of “Aldeia da Lagoa Branca” has the majority of its population classified as black.

## Discussion

Isolated Brazilian communities with a high frequency of people with albinism have been described in scientific and journalistic publications for decades. This work identified 13 clusters of albinism in the Northeast, Southeast and South of the country, and rumors to be confirmed in the North and Midwest. This finding reflects different aspects of the formation and organization of Brazilian society and contributes to the epidemiological characterization of albinism in Brazil, which still lacks so much information (Marçon et al., 2020; Brasil, 2021).

Inbreeding is one of the main risk factors associated with clusters of albinism in many of these cases, as Kromberg and Jenkins (1982) noted in indigenous communities in South Africa. One expected effect of inbreeding is the increase in homozygosity, which facilitates the manifestation of autosomal recessive hereditary diseases, such as albinism (Torres-Hernández et al., 2021). Due to sociocultural (e.g., cultural, religious beliefs) and economic reasons (e.g., land or property owning), consanguinity is not uncommon in human populations. It is estimated that couples related as second cousins or closer and their progeny account for an estimated 10.4% of the global population (Bittles and Black 2010; Romeo and Bittles, 2014). 

Inbreeding was suggested as the main reason for the high rates of albinism in the following genetic isolates in Brazil: Filús (AL) (Silva, 2015; Levy, 2019), Ilha dos Lençóis (MA) (Freire-Maia et al., 1978) and Aldeia Caruguá (PR) (Brembatti, 2015). Freire-Maia *et al.* (1978) observed 20% of consanguineous unions in Ilha de Lençóis (MA), even if the degree of kinship was not close. The founder effect, when a small group of individuals becomes isolated from a larger population leading to the increase of some rare allele frequencies associated with inbreeding, is the most possible explanation for the high frequency of albinism in most clusters here identified. 

Woolf (2005) reviewed the high prevalence of OCA2 in Native American communities (1:28 to 1:6,500) in southern Mexico, southwestern USA, eastern Panama and south Brazil. Although albinism implies the reduction of reproduction (Darwinian fitness), demographic characteristics allowed the increase in the frequency of occurrences of OCA in certain Amerindian populations throughout history (e.g., founder effect, genetic drift). In Brazil, protected both *quilombos* and indigenous protected lands are geographically limited, enhancing the isolation and inbreeding in these communities.

High prevalence rates of albinism have been reported in populations with African ancestry, with prevalence ranging from 1/5,000 to 1/15,000 in sub-Saharan Africa, including many reports about isolated, rural communities in some countries (Hong et al., 2006; Marçon et al., 2020). Lund et al. (2007) reported a high incidence of albino people in native communities in South Africa, which is considered a relatively common hereditary condition (Lund *et al.*, 2007; Marçon *et al.*, 2020). 

In this work, we identified four clusters of albinism in *quilombos* in Brazil, formed from the 16th century onwards due to the escape and isolation of enslaved people, mainly of African origin, and their descendants, many of whom remain in relative isolation until today (Cardoso-dos-Santos et al., 2020). Two of these clusters - the “Filús” Community in Santana do Mundaú (AL) and other in Quipapá (PE) - are geographically and culturally circumscribed in the region of “Quilombo dos Palmares”, the highest *quilombo* from Latin America. Both were also identified in the work of Cardoso-dos-Santos *et al.* (2020), who have crossed surname analysis with health and historical data to identify clusters of genetic diseases in Northeast Brazil. All clusters from Bahia (Ilha da Maré, Ilha dos Sapinhos, Miguel Calmon and Aldeia Lagoa Branca) present a high percentage of African ancestry, and some of them are geographically isolated (islands) (Moreira et al., 2016, 2019). 

OCA seems to be frequent in Kaingang communities, and there are reports back to 1930, as reviewed by Rodrigues (2021). In Rio Grande do Sul, a report of the health secretariat of 2014 refers to a number of 50 people registered with a diagnosis of albinism among 33,000 living on indigenous lands (18,000 Kaingang) (Rio Grande do Sul, 2015).

The majority of the clusters identified here were located in geographically isolated regions (e.g., islands, villages, quilombos and native communities) with severe situations of social and economic vulnerability. Residents with albinism on the Ilha da Maré (Salvador-BA), for example, have access to only one primary health unit on the island and need to travel to Salvador by boat to have access to ophthalmological and dermatological treatment (Pitombo and Spinassé, 2021). A similar situation is experienced by the inhabitants of Ilha de Sapinhos (Maraú-BA), whose only means available is a boat (Oliveira, 2005). This scenario of socio-economic precariousness and difficult access to health care is repeated in all communities covered in this study. Madeiro (2009) adds that the *quilombola* community Filús (Santana do Mundaú-AL) lived for decades with a lack of information about their civil rights, lower levels of education and higher rates of poverty, difficulty in accessing means of transport and communication. 

Another concern is the discrimination which is reported in some indigenous communities. Two publications described in detail the exclusion of social life and lack of prospect to constitute a family of individuals with albinism. Both of these publications were only case descriptions, one a newspaper report (Freitas *et al.*, 2005; Pinheiro, 1996).

The clusters presented here probably underrepresent many undetected or unreported communities. Our approach was systematic but was based only on written literature. However, it is important to mention that the search in the grey literature (news, websites, etc.) on the internet helped us confirm previous rumors in the CENISO and find unreported clusters and studies not published as formal articles in scientific journals. That could be a valuable strategy for other rare disorders or congenital anomalies. 

Rare conditions also usually have wide variations in prevalence, especially in small populations, as in some of the clusters described here. As expected, prevalences in communities with few residents had the highest estimated prevalences. We have to acknowledge that a sampling effect is possible. We couldn’t detect in the literature reviewed the pedigrees to check the number of siblings in one family or independent cases. We classified the three newspaper publications as unconfirmed clusters since they all referred to cases in one family (sibship). Another one was a case report only. We didn’t exclude them since all were in isolated indigenous communities and might reflect the presence of a pathogenic allele. 

## Concluding remarks

The present review identified 18 genetic isolates of albinism in Brazil. Of these isolates, eight are in the Northeast region, most in *quilombos* or areas with high levels of African ancestry. Six clusters come from Kaingang / Guarani native populations. Three clusters are located on islands (Ilhas da Maré, Sapinhos-BA, and Lençóis-MA). Among these occurrences, five are not-confirmed yet as clusters of albinism. Four of these non-confirmed reports belong to the original population: one in Acre (Kaxinawa), one in Mato Grosso (Kuikuro), one in São Paulo (Guarani), and one in Rio Grande do Sul (Kaingang). The last not-confirmed occurrence is from Baía Formosa (RN), which has a diverse ancestry. Brazilian clusters are often socio-economically vulnerable, which makes it difficult for people with albinism to access information and quality treatment. Although albinism has been described in Brazil for decades, little is known about the genetic architecture of this condition in our country and the origin of our founder mutations, especially in indigenous populations. We are aware of the limitations and sensible of the cultural differences concerning isolated communities and the native Brazilian people. However, we reinforce the need for a tailored approach to these communities, including appropriate medical care, social support, and genetic counselling whenever possible.
